# Post-varicella arterial ischemic stroke in children and neurocognitive performance: a 4-year follow-up study

**DOI:** 10.31744/einstein_journal/2022AO6360

**Published:** 2022-04-28

**Authors:** Regina Maria Rodrigues, Sylvia Costa Lima Farhat, Leandro Tavares Lucato, Tania Miyuki Shimoda Sakano, Paulo Scatulin Gerritsen Plaggert, Erasmo Barbante Casella, José Albino da Paz, Cláudio Schvartsman

**Affiliations:** 1 Hospital das Clínicas Faculdade de Medicina Universidade de São Paulo São Paulo SP Brazil Instituto da Criança, Hospital das Clínicas, Faculdade de Medicina, Universidade de São Paulo, São Paulo, SP, Brazil.; 2 Hospital das Clínicas Faculdade de Medicina Universidade de São Paulo São Paulo SP Brazil Hospital das Clínicas, Faculdade de Medicina, Universidade de São Paulo, São Paulo, SP, Brazil.; 3 Hospital Israelita Albert Einstein São Paulo SP Brazil Hospital Israelita Albert Einstein, São Paulo, SP, Brazil.

**Keywords:** Stroke, Basal ganglia cerebrovascular disease, Chickenpox, Child

## Abstract

**Objective:**

To analyze data from children who were previously healthy and presented with post-varicella arterial ischemic stroke upon arrival when admitted to the emergency room, with focus on the clinical/laboratory aspects, and neurocognitive performance after four-year follow-up.

**Methods:**

Seven children presenting with arterial ischemic stroke after varicella were evaluated at pediatric emergency services in the city of São Paulo (SP), Brazil. Ischemic stroke was determined by magnetic resonance imaging/magnetic resonance angiography in a topography compatible with the areas supplied by the middle cerebral or internal carotid arteries. IgG-class antibodies against varicella zoster virus and varicella-zoster virus DNA by polymerase chain reaction in cerebrospinal fluid were tested. Patients with prothrombotic conditions were excluded. The Pediatric Stroke Outcome Measure was applied upon admission and 4-years after the stroke.

**Results:**

All patients (age range: 1.3 to 4 years) included presented chickenpox 5.1 (±3.5) months before. All patients had analysis of anti-varicella-zoster-virus-IgG in cerebrospinal fluid, but only three (43%) had a positive result. Of the patients 43% had no vascular lesions identified in magnetic resonance angiography. All patients showed improvement in their sequela scores. After 4 years, five patients displayed good evolution in the Pediatric Stroke Outcome Measure, and only one patient presented with a score of 2 in the sensorimotor and cognition areas. No recurrence of arterial ischemic stroke was observed.

**Conclusion:**

We reinforced the non-progressive course of post-varicella arterial ischemic stroke after 4-year follow-up. The presence of varicella-zoster-virus-DNA detected by polymerase chain reaction, and/or intrathecal IgG antibody against varicella zoster virus, and angiopathy location in magnetic resonance angiography were not determining for the diagnosis. Invasive tests, with low sensitivity, should be well considered in the diagnosis of post-varicella arterial ischemic stroke.

## INTRODUCTION

Arteriopathy caused by viral infections is one of the most important mechanisms of arterial ischemic stroke (AIS) in the pediatric population. Several viruses were previously associated with AIS, such as the varicella-zoster virus (VZV). This is a DNA virus, like the herpes virus, which is able to remain latent in neuronal cells for months after primary infection. Therefore some complications, such as transient cerebral arteriopathy (TCA), may occur up to one year after varicella.^(1)^ Transient cerebral arteriopathy affects the middle cerebral artery (MCA) or the internal carotid artery (ICA), manifesting in the basal ganglia of children with symptoms of AIS.^(1)^ Post-varicella AIS, although rare, accounts for approximately 30% of all AIS in children.^(1)^

To date, there is conflicting evidence regarding the identification of antibodies against VZV, or VZV DNA in cerebrospinal fluid for diagnosis of post-varicella AIS in children.^(1,2-6)^

## OBJECTIVE

To analyze data from children who were previously healthy and presented with post-varicella arterial ischemic stroke to emergency room, and focused on the clinical/laboratory aspects and neurocognitive performance after 4-year follow-up.

## METHODS

This study reported a series of seven cases of patients admitted to the emergency room with post-varicella AIS and longitudinal follow-up. We evaluated charts of these patients from five different hospitals (*Instituto da Criança, Hospital Israelita Albert Einstein, Hospital Santa Catarina, Hospital Infantil Darcy Vargas* and *Hospital São Camilo*) in the city of São Paulo (SP), Brazil, from December 2013 to December 2017. This study was approved by the local Ethics Committee of *Hospital das Clínicas* of the *Faculdade de Medicina* of the *Universidade de São Paulo* (USP), CAAE: 43629315.2.1001.0068, protocol 1.036.344, and *Hospital Israelita Albert Einstein* (HIAE), CAAE: 43629315.2.3002.0071, protocol 1.343.515.

The inclusion criteria were chickenpox in the past 12 months, referred by parents or guardians, AIS determined by clinical examination and magnetic resonance imaging (MRI)/magnetic resonance angiography (MRA) in a topography compatible with the areas of the MCA or ICA, cerebrospinal fluid sample collected and analyzed, and aged under 18 years. A radiologist reviewed each MRI scan to confirm the presence of AIS. Magnetic resonance imaging and MRA were performed within 24 to 72 hours after the initial visit. All participating hospitals had 1.5 Tesla MRI scanners. Individuals who presented with other risk factors for stroke, such as prothrombotic conditions, cardiopathies, trauma or other cerebral arteriopathies were excluded. Laboratory tests were used to rule out prothrombotic conditions, and two-dimensional Doppler echocardiography was performed to rule out cardiopathies or cervical arterial diseases.

Results from the ELISA of cerebrospinal fluid to detect immunoglobulin G (IgG) antibodies against VZV, and polymerase chain reaction (VZV-DNA-PCR) to detect VZV virus DNA were collected up to 10 days after the onset of AIS in all patients.

Sensorimotor, language and cognitive sequalae were accessed by the Pediatric Stroke Outcome Measure (PSOM) upon admission and 4 years after AIS.^(7)^ Patients were evaluated by physical therapy, speech therapy, and psychology services for specific follow-up, after hospital discharge.

## RESULTS

Seven children were included in this study; five of them (71.4%) were male. The median age at stroke was 3.8 years (range: 1.3 to 4), and the mean period between the clinical presentation of AIS and the episode of chickenpox was 5.1 (± 3.5) months.

Table 1 includes a list of demographic parameters, clinical presentations, cerebrospinal fluid results (including IgG antibodies against VZV, and VZV-DNA-PCR), treatments received, and progression. All patients had an analysis of IgG against VZV in cerebrospinal fluid, but only 3 (43%) of them had a positive result.

**Table 1:** Demographic, clinical/laboratory parameters, and treatment for each patient admitted to the emergency room with post-varicella arterial ischemic stroke

Case	Age (years)	Sex	Months after onset of chickenpox	Varicella vaccine	Clinical presentation	CSF analysis	EEG	CSF VZV PCR-DNA	CSF Anti- VZV IgG	Angiopathy- location	Treatment	Evolution
1	2	Male	2	No	Complete and disproportionate right hemiparesis with aphasia since the day before. Strength *deficit* improved within 72 hours and aphasia within 24 hours	2 leukocytes/mm^3^, protein level and glucose level were normal	Normal	-	-	Normal	ASA	No *deficit*
2	4	Female	4	1 dose	Disproportionate right hemiparesis and left facial paralysis. The *deficit* showed progressive improvement, with complete recovery within 7 days	10 leukocytes/mm^3^ with 71% lymphocytes, 25% monocytes, 4% macrophages. Proteins: 0.10g/L. Glucose: 49mg/dL	Normal	-	+	Normal	ASA, acyclovir	No *deficit*
3	4	Male	1	No	2 left hemiparesis episodes within an interval of 2 days. First episode showed complete reversal. Second episode was accompanied by focal convulsive seizures, which resolved spontaneously. He showed no *deficits* after 10 days and no new episodes of AIS	5 leukocytes/mm^3^; proteins: 0.18g/L and glucose: 48mg/dL	Normal	NR	-	Normal	ASA	
4	4	Female	10	No	Complete and disproportionate right hemiparesis, with aphasia that improved after 6 days. No new episodes of AIS or neurocognitive sequela	4 leukocytes/mm^3^; proteins: 0.12g/L and glucose: 52mg/dl	Normal	-	-	MCA (M1, M2)	ASA	No *deficit*
5	1.3	Male	5	No	Complete and proportionate hemiparesis with progressive improvement of the motor *deficit* within 5 days, without new episodes of AIS	2 leukocytes/mm^3^ proteins: 0.2g/L and glucose: 42mg/dL	Disorganized background activity on the left hemisphere, without epileptiform activity	-	+	Distal ICA, A1, MCA (M1)	ASA, acyclovir, corticosteroids	Mild hemiparesis
6	3.8	Male	4	No	Gradual hemiparesis over a period of 2 days. Evolved with dysarthria and aphasia that resolved 3 days later	5 leukocytes/mm^3^; proteins: 0.10g/L and glucose: 56mg/dL	Normal	NR	+	Distal ICA, MCA (M1)	ASA	Mild hemiparesis
7	2	Male	1	No	Complete and proportioned right hemiplegia, associated with aphasia that improved partially after 10 days. He suffered focal convulsive seizures that evolved with generalization 36 hours after the onset of aphasia, which resolved with the use of phenytoin	25 leukocytes/mm^3^ with 65% lymphocytes, 25% monocytes; proteins: 0.43g/L and glucose: 56mg/dL	5 months later: disorganized background activity on the left hemisphere. Epileptiform activity projecting to the bilateral central and middle regions, with predominance to the left	-	-	MCA (M1, M2, M3)	ASA, acyclovir	Epilepsy, hemiparesis

CSF: cerebrospinal fluid; EEG: electroencephalogram; VZV: varicella-zoster virus; PCR-DNA: Polymerase Chain Reaction- Deoxyribonucleic acid; AIS: arterial ischemic stroke; ASA: acetylsalicylic acid; NR: not related; MCA: middle cerebral artery; ICA: internal carotid artery.

**Figure 1:**
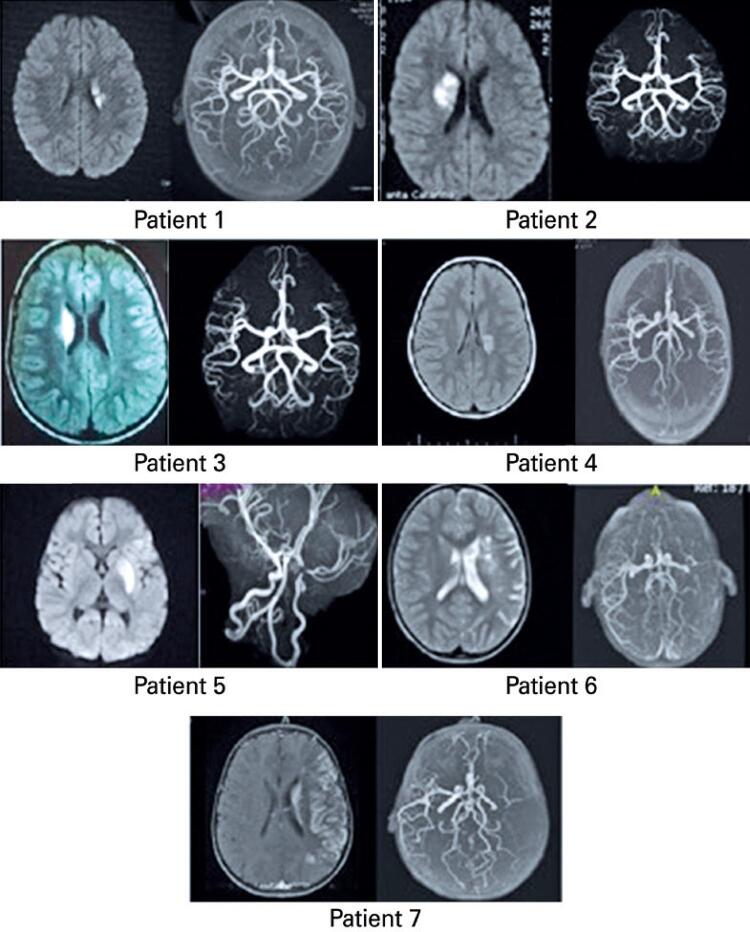
The brain magnetic resonance images from each of the seven patients with post-varicella arterial ischemic stroke

All patients were initially evaluated by computed tomography, and no signs of mineralizing angiopathy could be found. Figure 1 shows the brain MRI from each of the seven patients. Table 2 shows the description of MR images.

**Table 2:** Description of the brain magnetic resonance images from each of the seven patients

Patient-1	Acute infarction hyperintensity on axial diffusion-weighted image in the left caudate nucleus (A). Magnetic resonance angiography is normal (B)
Patient-2	Acute infarction hyperintensity on axial diffusion-weighted image in the basal ganglia on the right (A). Magnetic resonance angiography is normal (B)
Patient-3	Infarction hyperintensity on axial FLAIR image (A) in the right caudate nucleus. Magnetic resonance angiography is normal (B)
Patient-4	Infarction hyperintensity on axial FLAIR image (A) involving the periventricular white matter to the left. Magnetic resonance angiography (B) shows mild stenosis in the distal portion of the ipsilateral M1 segment
Patient-5	Acute infarction hyperintensity on axial diffusion-weighted image in the basal ganglia to the left (A). Magnetic resonance angiography (B) shows a severe stenosis in the top of the corresponding ICA, extending to the proximal segments of anterior (A1) and middle (M1) cerebral arteries
Patient-6	Chronic infarction hyperintensity on axial T2-weighted image (A) in the basal ganglia to the left; there is compensatory enlargement of the adjacent lateral ventricle and some cavities. Notice also volumetric changes involving almost the entire middle cerebral artery territory, characterized by enlarged sulci. Magnetic resonance angiography (B) shows a severe stenosis in the top of the corresponding ICA, extending to the M1 segment. There is poor characterization of the distal ramifications of this middle cerebral artery
Patient-7	Axial postcontrast T1-weighted image (A) depicts a subacute infarction involving almost the entire territory supplied by the middle cerebral artery. Magnetic resonance angiography (B) shows a severe stenosis in the top of the corresponding ICA, extending to the M1 segment, suggesting the possibility of occlusion / subocclusion of these vessels

ICA: internal carotid artery.

Four patients presented with unilateral vascular lesions in the MCA and/or ICA, while the MRI from three patients were normal. In five children, MRA was repeated after two to eight months to exclude progressive arteriopathy.

Table 3 shows the PSOM results of patients during treatment and 4 years later. After 4 years, four patients had a good progression, and scored zero on the PSOM. One patient had a score of 0.5, which is also considered good. Two patients evolved with scores greater than or equal to 1, corresponding to a moderate *deficit* with slowness of function. These patients had a greater degree of sensorimotor sequelae and were supported by neurology/psychology and physical therapy groups. One of these two patients presented with a score of 2 in the sensorimotor and cognition areas after 4 years. No recurrence of AIS was observed during the 4-year follow-up.

**Table 3:** Assessment of sensorimotor, language or cognitive sequelae using the Pediatric Stroke Outcome Measure(7)

Cases	Language production at AIS time/2018	Right sensorimotor at AIS time/2018	Left sensorimotor at AIS time/2018	Comprehension at AIS time/2018	Cognition at AIS time/2018	Score at the time of AIS onset	Final score in 2018
1	0/0	1.0/0	0/0	0/0	0/0	1.0	0
2	0/0	0/0	1.0/0	0/0	0/0	1.0	0
3	0/0	1/0.5	0/0	0/0	0/0	1.0	0
4	0/0	1.0/0	0/0	0/0	0/0	1.0	0
5	0.5/0	1.0/0.5	0/0	0/0	0/0	1.5	0.5
6	2.0/0	2.0/0.5	0/0	0/0	0.5/0.5	4.5	1.0
7	2.0/0	2.0/1.0	0/0	1.0/0	1.0/1.0	6.0	2.0

0: no *deficit*; 0.5: minimal *deficit* without functional consequence; 1: moderate *deficit* with slowing of function; 2: severe *deficit* with missing function. AIS: arterial ischemic stroke.

## DISCUSSION

In this study we found the presence of viral DNA and/or intrathecal IgG antibodies against VZV, and angiopathy location in MRA, were not determining for diagnosis of post-varicella AIS. Our study adds to previous literature by describing sensorimotor, language and cognitive sequelae evaluated by the PSOM, in a 4-year follow-up after AIS. Therefore, the need of invasive and low-sensitivity tests should be well considered in the diagnosis of post-varicella AIS.

Only five previous studies included at least four children each, with confirmed radiological imaging characteristic of ischemia in the territories of the MCA or ICA after an episode of varicella (Table 4).^(2-6)^

**Table 4:** Reports of series of cases (four or more children) evaluating children with post-varicella arterial ischemic, confirmed by magnetic resonance imaging and magnetic resonance angiography

Authors	Age	Months after varicella	PCR-cerebrospinal fluid	Anti-VZV IgG-cerebrospinal fluid	Sex	Angiopathy or localization	Treatment
Bartolini et al.^(2)^	3.6 years	0.5	NR	NR	Male	MCA	ASA
	2.2 years	1	NR	NR	Male	MCA (M1-M2)	ASA, heparin, antibiotics
	4.2 years	7	NR	NR	Female	Bilateral A1, left M1	ASA, valproic acid
	3 years	3	+	NR	Male	dICA, ACA, MCA	ASA, acyclovir
	2 years	2	-	NR	Male	dICA, ACA (M1-M3), MCA	ASA
Reis et al.^(3)^	22 months	10	NR	NR	Male	MCA	ASA, enoxaparin
	26 months	10	NR	NR	Female	NR	ASA
	4.5 years	1	NR	NR	Female	MCA (M1)	ASA
	10 months	2	NR	NR	Female	MCA (M1)	ASA
Dunkhase-Heinl et al.^(4)^	22 months	1	-	-	Female	MCA (M1)	Acyclovir, prednisone
	15 months	6	+	+	Male	MCA (M1)	Acyclovir, prednisone
	18 months	5	+	+	Male	MCA (M1)	Acyclovir, prednisone
	13 months	1.5	+	-	Female	ICA, MCA (M1)	Acyclovir, prednisone
	2 years	6	NR	NR	Male	ICA	NR
	3.5 years	3	NR	NR	Female	MCA	NR
	5 years	4	NR	NR	Female	MCA, ICA/ACA	NR
	3 years	4	-	NR	Male	MCA	NR
	8 years	6	-	NR	Female	MCA, ICA	NR
	3 years	2	NR	NR	Male	NR	NR
Science et al.^(5)^	3.5 years	3	_	NR	Male	NR	NR
	10 years	0.5	NR	NR	Female	MCA	NR
	11.5 years	6	_	NR	Female	MCA	NR
	9 years	1	NR	NR	Male	MCA	NR
Helmuth et al.^(6)^	2 years	3	NR	NR	Male	ACA	Acyclovir, methylprednisolone, ASA, Antibiotics
	6 years	1	+	-	Female	MCA (M1)	NR
	2 years	2	+	NR	Female	Normal	NR
	1 years	1	+	-	Female	MCA, ICA	NR
	4 years	8	+	+	Male	Normal	NR
	4 years	8	+	NR	Male	MCA (M2)	NR
	5 years	6	+	+	Male	ICA	NR
	5 years	5	+	+	Male	Basilar	NR
	5 years	8	+	NR	Male	MCA, ACA, ICA	NR
	5 years	4	+	-	Male	MCA, ACA, ICA	NR

PCR: polymerase chain reaction; IgG: immunoglobulin G; VZV: varicella-zoster virus; MCA: middle cerebral artery; ASA: acetylsalicylic acid; dICA: distal internal carotid artery; ACA: anterior cerebral artery; NR: not related; ICA: internal carotid artery.

Of these, only two^(2,6)^ analyzed the cerebrospinal fluid to look for IgG antibodies against VZV; only five of ten children tested had a positive result for IgG antibodies against VZV. In our study, all patients had an analysis of IgG antibodies against VZV in cerebrospinal fluid and only three (43%) of them had a positive result. Some authors have suggested that, in location where there is universal varicella immunization, tests for IgG antibodies against VZV should be more sensitive than commercial VZV ELISA,^(8)^which could help explain our anti-VZV IgG detection rates slightly lower than found in previous studies (43% *versus* 50%). However, given the small sample size, this difference could be explained by variance in the prevalence estimate, and further studies with larger samples are required to clarify this issue. Regarding varicella immunization, there is no association between AIS and live attenuated VZV of varicella vaccine, which has been recommended since 1996. Donahue et al.^(9)^ assessed 3.2 million children, and 1.14 million of them received varicella vaccine in the United States. The authors found varicella vaccine presented no association with increased risk of AIS. In our study, only one patient received one dose of VZV vaccine, probably because in Brazil it was included in the public immunization program only in 2013.

In our study, all children tested had negative results for VZV-DNA-PCR. Helmuth et al.^(6)^ identified previously healthy children with AIS, who had a recent history of varicella infection prior to the neurological condition. Nine out of 13 individuals tested had positive results for cerebrospinal fluid-VZV-DNA-PCR. Miravet et al.^(10)^ reported results similar to ours, evaluating children with and without comorbidities. These conflicting findings could be the result of the small sample sizes included; nonetheless, the absence of anti-VZV antibodies in the cerebrospinal fluid does not rule out the diagnosis in children who meet the criteria for post-varicella AIS.^(1,6,11)^

The main limitation of the present study was the fact it is a series of cases based on data collection from medical records. However, there are only two reports that included more than seven patients with post-varicella AIS, confirmed by MRI and MRA.

Our study reinforces the importance of assessing the approach to diagnose post-varicella AIS, which considers the clinical presentation, history of varicella, neuroimaging findings and, possibly, anti-VZV antibodies in the cerebrospinal fluid. It should also be noted that MRA was unable to rule out the diagnosis, since we observed three patients whose arterial lesions could not be located by MRA, a finding that had been described previously.^(6)^ Other diagnostic techniques, such as conventional arteriography, might be more accurate, but are probably very invasive for routine practice.

Our study highlights the nonprogressive character of post-varicella arteriopathy by the PSOM, in the 4-year follow-up. It has been used to better determine the degree of neurological impairment following AIS over time, since the brain of children is still developing, unlike of adults.^(7)^ After 4 years, the sensorimotor sequelae were more often observed than cognitive and language ones, which reflects the fact post-varicella AIS is mainly subcortical. Additionally, none of our patients presented with new ischemic or hemorrhagic episodes, reinforcing its monophasic course.

Rehabilitation therapy with neuropsychology, physical therapy and speech therapy may improve the outcomes of post-stroke patients, but these therapies have to be more structured in pediatrics. Regarding treatment, all patients received ASA, and two patients in whom intrathecal antibodies were identified received acyclovir. Treatment with acyclovir may be justified by the finding of VZV DNA in the arteries of a child, who died due to post-varicella AIS.^(8)^ However, it remains unclear whether acyclovir could prevent recurrence in healthy children.^(8)^ Only one patient in this series received corticosteroids. Patients who were not treated with antivirals or corticosteroids showed no higher risk of neurological impairment than those who were; similar results were found by Bartolini et al.^(2)^

## CONCLUSION

In this study, we reinforced the nonprogressive course of post-varicella arterial ischemic stroke after 4 years of follow-up. The presence of viral DNA and/or intrathecal IgG antibodies against varicella-zoster virus, and angiopathy location in magnetic resonance angiography, were not determining for diagnosis of post-varicella arterial ischemic stroke.

In addition, the necessity of invasive and low-sensitivity tests should be well considered in the diagnosis of post-varicella arterial ischemic stroke.
